# Fighting Coronavirus One Personality at a Time: Need for Structure, Trait Victimhood, and Adherence to COVID-19 Health Guidelines

**DOI:** 10.3389/fpsyg.2020.576450

**Published:** 2020-11-30

**Authors:** Yossi Maaravi, Boaz Hameiri, Tamar Gur

**Affiliations:** ^1^Interdisciplinary Center, The Adelson School of Entrepreneurship, Herzliya, Israel; ^2^The Evens Program in Conflict Resolution and Mediation, Tel Aviv University, Tel Aviv-Yafo, Israel; ^3^Psychology Department, Hebrew University, Jerusalem, Israel; ^4^Interdisciplinary Center, School of Psychology, Herzliya, Israel

**Keywords:** coronavirus, COVID-19, mental rigidity, need for closure, need for structure, victimhood, self-efficacy, adherence

## Abstract

Following the outbreak of the COVID-19 pandemic, authorities have issued several guidelines to curb the pandemic's disastrous effects. However, measures' effectiveness is dependent upon people's adherence to them. Therefore, it is crucial to understand the potential factors that explain guideline adherence. In the present brief research report, we investigated need for structure and trait victimhood, i.e., the tendency to feel like a victim, and their effect on fear of the pandemic, which in turn, predicted guideline adherence. Furthermore, the association between fear and guideline adherence was shaped by participants' global self-efficacy: higher levels of self-efficacy predicted more guideline adherence regardless of fear levels. The present findings may be relevant to health messaging endeavors aiming to improve compliance with guidelines.

## Introduction

In late 2019, the first cases of COVID-19 were reported in Wuhan, China (Guan et al., 2020). In just a few months this virus had spread across the globe, infected millions, killed hundreds of thousands and caused trillions of dollars in damages to the world economy (Ayittey et al., 2020). Consequently, the World Health Organization (WHO) declared COVID-19 a pandemic and announced a global emergency (Sohrabi et al., 2020). Health authorities and governments globally have responded by issuing numerous guidelines (Lau et al., 2020), the most common of which were: isolation, quarantine, and social distancing (Wilder-Smith and Freedman, 2020). Guidelines for personal measures included sterilizing surfaces, wearing masks, washing hands, etc. (Zhou et al., 2020).

The success of such efforts relies on adherence to these measures. Since some people who are able to adhere to the guidelines, do so, while others do not, adherence is possibly affected by various personal factors. For example, Zhong et al. (2020) have recently demonstrated that adherence to preventive measures depended on people's differential knowledge and attitudes toward COVID-19. Other possible explanations might include solution aversion (Campbell and Kay, 2014), resistance to scientific information (Hornsey and Fielding, 2017), and—as shown regarding COVID-19—even political orientation (Pennycook et al., 2020).

One critical category of personal factors that has been repeatedly shown to affect health-related behaviors and specifically adherence to health guidelines is personality (e.g., Christensen and Smith, 1995). Some notable examples are the lack of adherence of implosive patients to asthma control guidelines (Axelsson et al., 2009) and of patients suffering from anxiety to multiple sclerosis disease-modifying therapies (Bruce et al., 2010). More relevant to the current article, Bogg and Milad (2020) have recently shown that adherence to COVID-19 guidelines was positively related to the personality trait of conscientiousness.

## The Present Study

In the current brief research report, we focus on two personality traits: mental rigidity (e.g., Webster and Kruglanski, 1994), and victimhood (Gabay et al., 2020a,b). As detailed, these traits are highly relevant due to the specific psychological characteristics of pandemic: uncertainty (Eichenberger et al., 2020) and fear (Ren et al., 2020).

### Mental Rigidity and Stressful Events

Mental rigidity received different names (such as need for closure, need for structure, tolerance of ambiguity, certainty orientation), each with its' own theoretical focus and variations (e.g., Webster and Kruglanski, 1994; Bar-Tal et al., 1997; Leone et al., 1999; Muluk and Sumaktoyo, 2010). Mental rigidity is the desire to reduce ambiguity through category-based processing and receive answers on given topics (Webster and Kruglanski, 1994). It leads to seeking simplified, one-sided information while disregarding more complex aspects of the situation (Sharifi, 2019), and psychological maladjustment to new situations (Kashima et al., 2017). This trait is of high relevance to stressful or uncertain circumstances, such as war, natural disasters, or disease (Kruglanski et al., 2002). Given the uncertainty around COVID-19 as a new virus with unprecedented spread, no vaccine, and dramatic consequences and measures, people high in mental rigidity may be more fearful than others (Webster and Kruglanski, 1994). Moreover, increased fear (Ren et al., 2020) should result in a greater tendency to follow health instructions, which may be perceived as a means to reduce uncertainty (Kruglanski et al., 2006). Mental rigidity was also related to reduced risk taking in various domains (Schumpe et al., 2017). A study conducted in the US during the COVID-19 pandemic reviled that anxiety related behavior patterns (e.g., stockpiling food) of mentally rigid people were greatly affected in such times of crisis (Brizi and Biraglia, 2020).

### Victimhood and Dealing With Life's Misfortunes

Trait victimhood is defined as “an ongoing feeling that the self is a victim … generalized across many relationships, such that victimization becomes a central part of the individual's identity.” (Gabay et al., 2020a, p. 361). Victimhood fundamentally affects emotions, cognitions, and behaviors (Gabay et al., 2020b). Particularly relevant to this research, Gabay et al. (2020b) found that individuals with high levels of trait victimhood were more likely to interpret ambiguous situations as threatening through a black-and-white prism (Gabay et al., 2020b; see also Schori-Eyal et al., 2017). We argue that this recently introduced personality trait may offer a simple, yet powerful, measure of individual differences when facing hardships and misfortunes. Specifically, this trait is relevant to the behavioral guidelines of the pandemic since individuals high in victimhood are hyper-vigilant, neurotic, and susceptible to threat, which should increase their level of fear. Such fear may lead to a greater tendency to follow health instructions (Ren et al., 2020). Nevertheless, the actual ability to follow the instructions, or lack thereof, can be captured by the concept of self-efficacy.

### Self-Efficacy and Adherence to Medical Instructions

The construct of self-efficacy was introduced by Bandura (1986) who defined it as “people's judgments of their capabilities to organize and execute courses of action required to attain designated types of performances” (p. 391). Self-efficacy beliefs determine how people feel, think, motivate themselves and behave. Though self-efficacy was mainly conceptualized as domain-specific, scholars have also suggested global self-efficacy (GSE) to describe people's global belief in their ability to cope with different challenges or uncertainties (Schwarzer et al., 1999). Bandura (1997) treated self-efficacy as the main factor in performance, postulating that motivation is affected by self-efficacy via goals selection. However, some argued that motivation, rather than self-efficacy, is central in determining future performance (Vancouver et al., 2001), and yet others postulated that the effect of self-efficacy varies along different levels of performance (Gur and Bar-Tal, under review).

We argue that GSE is relevant to the current endeavor as it captures one's perceived competence to effectively cope with challenging and stressful situations (Schwarzer et al., 1999; Judge and Bono, 2001). Indeed, self-efficacy was shown relevant to coping with health and medical situations. Specifically, past research has described how GSE affects adherence to medical instructions and health guidelines across various settings, ranging from dietary adherence (Warziski et al., 2008), to HIV medication adherence (Wolf et al., 2007). Since increased fear was found to motivate adaptive danger control actions (Witte and Allen, 2000), in the case of COVID-19, fear may represent one's strength of motivation to adhere to health guidelines. Therefore, we predicted that it may interact with GSE in affecting adherence to guidelines. Thus, we hypothesized that while fear should mediate the relationship between mental rigidity and trait victimhood and adherence to COVID-19 health guidelines, GSE would moderate the extent to which fear would in fact translate into actual adherence.

To summarize, the current research focuses on the relationship between mental rigidity, operationalized using the Need for Structure (NFS) scale, and trait victimhood, and adherence to COVID-19 health guidelines. This is based on previous research showing that mentally rigid individuals tend to be more fearful (Webster and Kruglanski, 1994), and that high trait victimhood individuals tend to mistrust others (Gabay et al., 2020b), which may also increase fear in the context of a pandemic. We hypothesized that mental rigidity and trait victimhood would be positively related to COVID-19 guideline adherence, and that fear would mediate these associations. Additionally, we hypothesized that GSE would moderate the extent to which fear would lead to guideline adherence, such that high- (vs. low-) GSE individuals would adhere more to the guidelines.

## Method

### Sample and Procedure

Three hundred and fifty four Israelis (48.6% women; *M*_age_ = 41.71, SD = 16.02) reported their mental rigidity (NFS), trait victimhood, GSE, fear of the coronavirus (COVID fear) and adherence to Israel's health department regulations regarding protection from the virus. Participants were recruited through an Israeli survey company (Midgam Project)^1^, responded electronically via the internet and were paid for their participation in the study. The data was collected from March 22nd until March 23rd, 3 days after emergency regulations were initiated in Israel (on March 19th) and 2 days after the first documented COVID-19 death in Israel (on March 20th)^2^. The study was reviewed and approved by the Interdisciplinary Center Herzliya Institutional Review Board (see ethics statement). All subjects provided informed consent to participate in the study. To protect the respondents' privacy, the survey was conducted anonymously.

### Measures

The presentation order of all scales and statements within them were randomized and all used a 7-point Likert-type scale ranging from “strongly disagree” (1) to “strongly agree” (7). We used the *NFS* scale to assess mental rigidity, since it has a short, validated, 11-item Hebrew version (Bar-Tal et al., 1997; α = 0.82). The *Trait victimhood* scale used an abridged version, consisting of nine statements (α = 0.82), of the scale developed by Gabay et al. (2020b). *COVID fear* was assessed by five statements, two of which (i.e., “I am very worried about being infected or infecting others that are close to me” and “I am afraid of corona disease”) were adapted from a fear of cancer scale (Vrinten et al., 2015). We developed the three remaining statements for the purposes of the current research. They refer to a unique aspect of epidemics vs. other disease, i.e., the risk to oneself, to one's closest environment, and to the society in general (α = 0.71). The *GSE* scale used a modified version of Zeidner et al. (1993) Hebrew GSE 10-item scale (Weber et al., 2013; α = 0.91). *Guideline adherence* was assessed using two items we developed for the current study (i.e., “*I make sure to wash my hands more often than I did before the coronavirus outbreak”;* and “*Since the coronavirus outbreak, I have been very careful to follow instructions (stay at home, reduce contact with people as much as possible, sneeze and cough into my elbow or tissue paper)”; r* = 0.43, *p* < 0.001)^3^.

## Results

For all variable means, SDs and correlations see Table 1. We tested our hypothesized moderated mediation path model, reasoning that our independent variables, i.e., NFS and trait victimhood, would lead to COVID fear, which in turn would lead to more guideline adherence; and that the association between COVID fear and guideline adherence would be moderated by GSE. We conducted a path analysis using Hayes's (2018) PROCESS (Model 14) bootstrapping command with 5,000 iterations controlling for participants age and gender^4^. Given that the PROCESS add-on cannot estimate a moderated mediation model with two parallel independent variables, we ran two models separately^5^: (1) indirect effect of NFS on guideline adherence through COVID fear, moderated by GSE, controlling for trait victimhood; and (2) the same model with trait victimhood as the independent variable, controlling for NFS.

**Table 1:** Means, SDs and correlations of all variables.

	**Mean (SD)**	**1**	**2**	**3**	**4**	**5**	**6**	**7**
Guideline adherence	5.99 (1.03)	–						
COVID fear	4.92 (1.13)	0.44^***^	–					
Need for structure	4.88 (0.88)	0.20^***^	0.30^***^	–				
Trait victimhood	4.54 (0.99)	0.20^***^	0.25^***^	0.39^***^	–			
Global self-efficacy	5.33 (0.85)	0.25^***^	0.01	−0.06	0.14^**^	–		
Age	41.71 (16.02)	0.06	0.10	−0.04	0.003	0.01	–	
Gender (1 = M, 2 = F)	–	0.13^*^	0.09	0.15^**^	0.09	0.04	0.02	–

* *p < 0.05*,

** *p < 0.01*,

*** *p < 0.001*.

**Figure 1:**
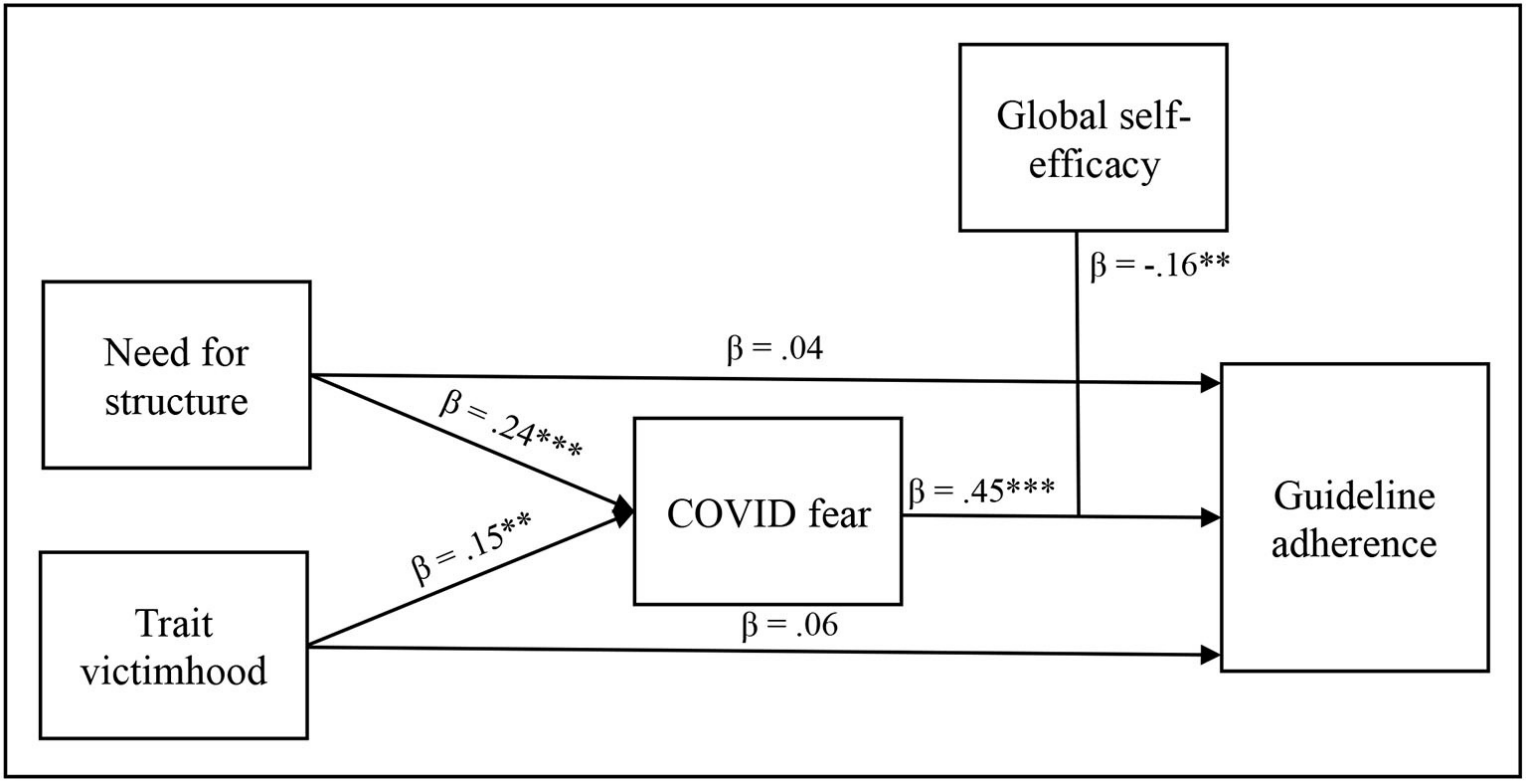
A path analysis of the moderated mediation of need for structure and Trait victimhood on guideline adherence through COVID fear, moderated by global self-efficacy. **p < 0.01, ***p < 0.001.

Both models with either NFS or trait victimhood as the independent variables yielded similar pattern of results. Specifically, both NFS and trait victimhood's total effects on guideline adherence (see Table 1) were no longer significant when the mediator, COVID fear, and its interaction with GSE were introduced into the models (β = 0.04, *SE* = 0.05, *p* = 0.428, 95% CI [−0.06, 0.15], and β = 0.06, *SE* = 0.05, *p* = 0.266, 95% CI [−0.04, 0.16], respectively). Guideline adherence was predicted by COVID fear (β = 0.45, *SE* = 0.05, *p* < 0.001, 95% CI [0.35, 0.55]) and by the COVID fear X GSE interaction (β = −0.16, *SE* = 0.05, *p* = 0.001, 95% CI [−0.25, −0.06]; see Figure 1).

To probe the interaction, we used simple slope analysis (Aiken and West, 1991). GSE was fixed at 1 SD below the mean, corresponding to low-GSE participants, and 1 SD above the mean, corresponding to high-GSE participants. Conditional effects showed that for low-GSE participants, COVID fear significantly predicted guideline adherence (β = 0.61, *SE* = 0.08, *p* < 0.001, 95% CI [0.45, 0.76]); while for high-GSE participants the effect of COVID fear on guideline adherence was still significant, but considerably smaller (β = 0.29, *SE* = 0.06, *p* < 0.001, 95% CI [0.17, 0.42])^6^.

**Figure 2:**
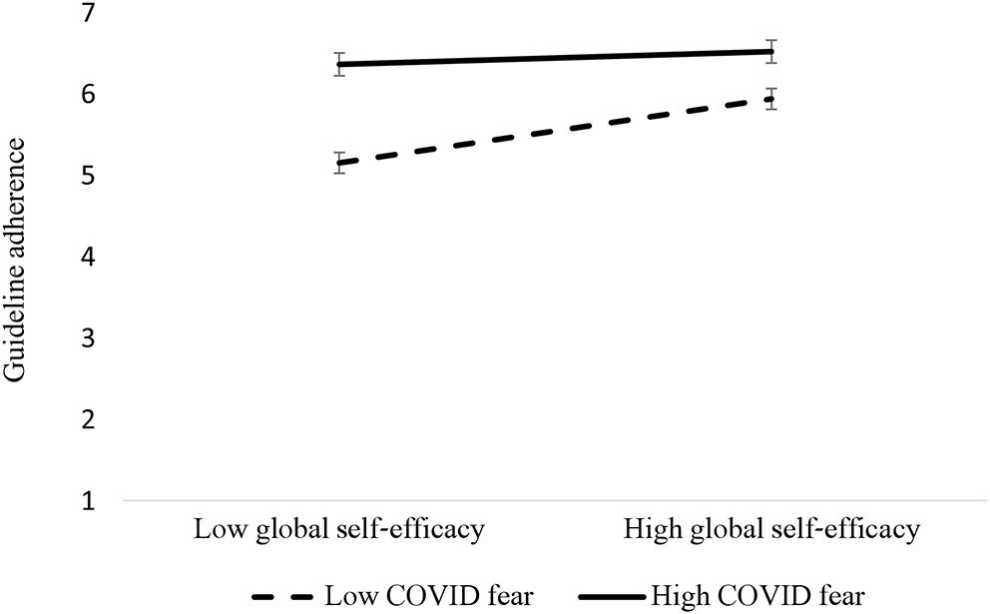
Guideline adherence as a function of the Interaction between COVID fear and global self-efficacy. Error bars represent 95% CIs.

Finally, the COVID fear X GSE interaction yielded a significant indirect effect via COVID fear for low-GSE participants in both the NFS and trait victimhood models (β = 0.15, *SE* = 0.04, 95% CI [0.07, 0.22], and (β = 0.09, *SE* = 0.04, 95% CI [0.02, 0.17], respectively). The indirect effect via COVID fear was still significant for high-GSE participants in both the NFS and trait victimhood models (β = 0.07, *SE* = 0.02, 95% CI [0.03, 0.12], and β = 0.04, *SE* = 0.02, 95% CI [0.01, 0.08], respectively), but considerably smaller, yielding both moderated mediation models to be significant (*index* = −0.04, *SE* = 0.02, 95% CI [−0.07, −0.01], and *index* = −0.02, *SE* = 0.01, 95% CI [−0.05, −0.002], respectively; see Figure 2).

## Discussion

In the current brief research report, we suggested that adherence to authorities COVID-19 health guidelines is associated with mental rigidity, operationalized as NFS, and trait victimhood. Our model suggests that these traits may be associated with guideline adherence through an indirect effect of fear of the pandemic. However, the extent to which fear predicted guideline adherence was moderated by GSE, such that for low-GSE, the extent to which participants feared the pandemic had a significantly bigger effect on guideline adherence, compared to the high-GSE participants.

Adherence to the COVID-19 health guidelines is dependent upon various factors. In some cases, adherence to guidelines issued by the authorities is predominately dependent upon the ability to do so, such as those considered emergency workers. Some cannot afford not to work if they do not get paid sick leave. And in other cases, people cannot properly practice social distancing if they live in densely populated neighborhoods (e.g., Bouie, 2020). For those who *are* able to adhere to the guidelines, we argue and show evidence that specific personality traits may be important factors affecting adherence.

Considering mental rigidity, we argue that a pandemic of such magnitude involves great uncertainty and fear for those high in mental rigidity, and thus following the guidelines might reduce this uncertainty (Kruglanski et al., 2006). Regarding trait victimhood, people high in this tendency may be fearful of the pandemic, due to their general tendency to be vigilant to potential harm. This, in turn, should lead to more adherence to hand washing and practicing social distancing due to high-victimhood individuals' self-reliance in protecting themselves, as well as their perceptions of moral superiority (Gabay et al., 2020a,b).

Interestingly, while victimhood has mostly been shown to lead to negative consequences, especially in the realm of interpersonal and intergroup relations (e.g., Gabay et al., 2020a; Vollhardt, 2020), the current research suggests it might also have positive outcomes for the individual and possibly for the community. Furthermore, unexpectedly, we found that trait victimhood was positively associated with GSE. Although trait victimhood should be distinguished from actual experienced victimization, which is characterized perception of powerlessness, this finding is interesting and should be examined in future studies.

In terms of possible implications of the current research, due to the great importance of pandemic related messages, they should be perfected to lead to favorable results. In today's world, in which so many messages are imparted via social networks, social marketers trying to promote more COVID-19 related guideline adherence can enhance their messages' effectiveness using tailored, or personalized messaging (e.g., Hirsh et al., 2012; Halperin and Schori-Eyal, 2020). Indeed, the literature on attitude change has long established that the effectiveness of a message is not only based on the message itself, but also on the message source, the medium, and the characteristics of the message recipient (Hovland et al., 1953; Greenwald, 1968). Yet, while most research has focused on the different messages used (e.g., Maaravi et al., 2011), differential characteristics of recipients may also influence the results of the persuasion attempt. The current research provides preliminary indications that individuals with high levels of mental rigidity and/or trait victimhood, might be more susceptible to messages that describe the risk of the COVID-19 pandemic, which might increase their compliance with the guidelines. Interestingly, we found that age, which has played a pivotal role in the deadliness of the pandemic (e.g., Remuzzi and Remuzzi, 2020), as well as with guideline adherence (Bogg and Milad, 2020), was neither significantly correlated with COVID fear nor with guideline adherence. This suggests that perhaps messaging that does not rely on emphasizing the potential risk could resonate more with older message recipients.

At this point, it should also be noted that the hypothesized independent variables in our model are personality traits, which in theory should precede and predict a response to a current event. Yet, the current research is correlational, and we cannot draw any firm conclusions regarding the causal relationships between the variables. Future research should attempt to establish causality by, for example, priming of a sense of victimhood (e.g., Baumeister et al., 1990) and then exploring its effects on fear and guideline adherence.

## Conclusion

In conclusion, the current report has pointed to the possible role mental rigidity, trait victimhood, and GSE play in fearing the COVID-19 pandemic, as well as in adhering to the guidelines issued by the authorities. Our model suggested that mental rigidity and trait victimhood both predicted fear of COVID-19, which in turn translated into more guideline adherence. High levels of fear yielded high levels of guideline adherence. However, when fear was not particularly high, participants' GSE was also associated with more guideline adherence. We argue that the current research may contribute to our understanding of how personality traits shape responses to adversities, as well as to the development of more effective messaging to promote message recipients' compliance with guidelines issued by the authorities.

## Data Availability Statement

The data that support the findings of this study are openly available at https://osf.io/rqbxu/?view_only=11b5049cb83943deaf87d01c448b2540.

## Ethics Statement

The studies involving human participants were reviewed and approved by the Institutional Review Board, IDC Herzliya Adelson School of Entrepreneurship (IRB #YMCD19003). All subjects provided informed consent to participate in the study. To protect the respondents' privacy, the survey was conducted anonymously. All procedures were in accordance with the Declaration of Helsinki.

## Author Contributions

YM: study design, data interpretation, writing, and literature search. BH: study design, data interpretation, writing, and figures. TG: data collection, data analysis, writing results, and figures. All authors contributed to the article and approved the submitted version.

## Conflict of Interest

The authors declare that the research was conducted in the absence of any commercial or financial relationships that could be construed as a potential conflict of interest.

## Footnotes

^1^We have chosen to use computerized questionnaire via a web based panel since past research indicated that online samples provide similar results to face-to-face samples, however, online samples tend to be more diverse (Casler et al., 2013). Midgam Project web panel was chosen as it includes over 50,000 panellists aged 17 years and older in Israel. It is one of the biggest panels in Israel, allowing its clients a representative sample of the population. It is highly regarded and used extensively for academic research and political surveys. Other variables (irrelevant to the current analysis) also collected in this survey will be published in a separate paper.

^2^On March 19th the Israeli government issued a stay-at-home order, limiting travel and work, except for essential needs such as getting food, medicines and medical or essential services, assistance to others in need, religious reasons, demonstration, blood donations and solitary sports.

^3^We omitted an additional item from this scale (i.e., “Adhering to the public guidelines is pointless, since we have no control over the disease's outbreak.”), since we realized that it did not directly address guideline adherence, and it also reduced the reliability of the scale.

^4^Since the study was cross-sectional, we controlled for age and gender to eliminate potential alternative explanations. The same pattern of results appears when not controlling for participants' age and gender.

^5^The two conducted PROCESS analyses represent one hierarchical regression, in which need for structure and trait victimhood are in the first step; COVID fear, general self- efficacy and the interaction between them are in the second step; and guideline adherence is the result variable. To receive the indirect effects for both IV's, we used the PROCESS add on. However, due to a technical limitation, the PROCESS add-on can calculate the indirect effect for only one IV at a time. To overcome this limitation, we conducted two analyses: in the first, trait-victimhood was the IV while need-for-structure was controlled for; and in the second need-for-structure was the IV while trait-victimhood was controlled for.

^6^Examining the interaction between COVID fear and GSE with COVID fear fixed at 1 SD below and above the mean indicated that for low fear participants GSE significantly predicted guideline adherence (β = 0.39, *SE* = 0.07, *p* < 0.001, 95% CI [0.26, 0.52]); but not for high fear participants (β = 0.08, *SE* = 0.07, *p* = 0.274, 95% CI [−0.06, 0.22]).
